# A fluffy nanofiber scaffold-based microenvironment enables a simplified, biology-centric scale-up strategy for mesenchymal stem/stromal cell culture

**DOI:** 10.3389/fbioe.2026.1808384

**Published:** 2026-04-14

**Authors:** Masashi Iwakami, Gabrielle Erwin Gemeniano Awitan, Riku Yamamoto, Masahiro Kino-oka

**Affiliations:** 1 Department of Biotechnology, Graduate School of Engineering, The University of Osaka, Osaka, Japan; 2 Biological Research Laboratories, Nissan Chemical Corporation, Saitama, Japan; 3 Research Base for Cell Manufacturability, Graduate School of Engineering, The University of Osaka, Osaka, Japan

**Keywords:** fluffy scaffold, mesenchymal stem/stromal cells, nanofiber, scaffold agglomeration, scale-up, suspension culture

## Abstract

Scalable suspension culture technologies are essential for the large-scale manufacturing of mesenchymal stem/stromal cells (MSCs). However, conventional microcarrier-based systems often fail to achieve scalable efficiency because cell–microcarrier dynamics vary across scales, necessitating complex, scale-dependent agitation protocols. To overcome this limitation, we designed a scale-up-oriented suspension culture system employing fluffy, fibrillated nanofiber scaffolds composed of chitosan and chitin. These nanofibers were readily suspended under continuous, gentle agitation and trapped cells on their surfaces, while spontaneously forming fluffy cell–scaffold aggregates through scaffold agglomeration. The low-adhesive chitosan nanofibers acted as physical spacers that prevented aggregate coalescence, thereby maintaining a microenvironment favorable for proliferation. By defining cell–scaffold aggregate size as the key scaling parameter—a biology-centric approach—, we successfully achieved scale-up from 30 mL to 5 L under continuous gentle agitation, yielding comparable specific growth rates of (3.66 ± 0.28) × 10^−2^ h^-1^ (30 mL), (3.27 ± 0.40) × 10^−2^ h^-1^ (1 L), and 3.50 × 10^−2^ h^-1^ (5 L), and reaching a total yield of 4.23 × 10^9^ cells in the 5-L bioreactor (a single run). These findings demonstrate that fluffy nanofiber scaffolds enable a scale-up strategy that reproduces the cellular microenvironment in a manner that is less affected by scale-dependent physical forces. This concept provides a new framework for designing scalable culture environments applicable not only to cell therapy manufacturing but also to culture supernatant production and cell–based food materials.

## 1 Introduction

Mesenchymal stem/stromal cell (MSC)-based therapies have gained increasing attention in regenerative medicine (Fernández-Garza et al., 2023). Current manufacturing of MSCs for transplantation mainly relies on two-dimensional (2D) culture systems that require numerous manual operations, thereby increasing the risk of contamination (Silva Couto et al., 2020). To reduce the number of manual operations associated with multiple vessels, multilayer flasks are often used to expand the available surface area per vessel and thereby increase cell yield (Rafiq et al., 2013). However, producing a sufficient number of cells remains particularly challenging when manufacturing allogeneic cell therapy products for multiple patients, each requiring approximately 10^6^ to 10^9^ cells per dose (Simaria et al., 2014; Tavassoli et al., 2018). Thus, scalable and operationally efficient three-dimensional (3D) suspension culture systems are required to reduce manual handling and enable the production of large numbers of cells (Simaria et al., 2014; Tavassoli et al., 2018).

3D suspension culture systems using stirred bioreactors are widely adopted for the production of biopharmaceuticals using microbial and animal cells. During scale-up, agitation becomes a key operational parameter for achieving comparable culture efficiency across scales. Agitation intensity is commonly defined by physical parameters such as power input per unit volume (P/V) or impeller tip speed, both of which influence mass transfer (Catapano et al., 2009). In microbial cultures, dissolved oxygen often limits growth; thus, agitation is tuned to maintain a comparable volumetric oxygen transfer coefficient (*k*_L_a) across scales. In animal cell culture, however, not only oxygen transfer but also shear stress generated by agitation critically affects the behavior of both anchorage-independent and anchorage-dependent cells because of their sensitivity to mechanical forces (Frisch and Screaton, 2001). For anchorage-dependent cells in particular, proliferation is tightly coupled with morphological dynamics—adhesion, aggregation, and collapse—that evolve continuously from seeding to expansion. Because these dynamics cannot be fully controlled by agitation alone, additional scale-up strategies are necessary.

In MSC suspension culture, which involves anchorage-dependent cells, either aggregate formation or adhesion to microcarriers is indispensable. When cells form aggregates solely through cell–cell adhesion, efficient expansion becomes difficult (Sart et al., 2014). Consequently, microcarriers that provide adhesive surfaces have been widely utilized (Silva Couto et al., 2020; Tsai et al., 2020). Yet, cell adhesion to microcarriers is influenced by multiple factors, including agitation-induced dispersion (Moreira et al., 2020), medium composition (Forestell et al., 1992; Yuan et al., 2014; Jorgenson et al., 2018), and pH (Yuan et al., 2014). Even after successful adhesion, microcarrier aggregation during proliferation frequently occurs (Zhang et al., 2023) depending on agitation conditions (Jossen et al., 2018), which can lead to reduced cell density (Zhang et al., 2023) and increased heterogeneity (Tsai et al., 2020). These issues highlight that conventional microcarrier-based suspension cultures face several agitation-related technical limitations, underscoring the need for novel design strategies to overcome them.

To address these challenges, we explored the use of nanofibers as scaffold materials in suspension culture systems. Nanofibers are fibrous materials with nanometer-scale diameters (Pei et al., 2021) whose bundled structures mimic the extracellular matrix (ECM) *in vivo*, making them attractive as cell culture surfaces (Beachley and Wen, 2010). Their bulky yet highly porous nature provides a large adhesive surface area per unit volume (Beachley and Wen, 2010), enabling high-density culture. For dynamic suspension culture, achieving good dispersibility is also essential. In this context, chitosan and chitin nanofibers were selected because of their favorable processability. Chitin, the second most abundant natural polysaccharide on Earth, forms the exoskeleton of crustaceans and insects (Pei et al., 2021), whereas chitosan is a deacetylated derivative of chitin (Croisier and Jérôme, 2013). Crab shells, often regarded as industrial waste, have recently been explored as renewable raw materials for nanofiber production (Ifuku et al., 2010). Previous studies have shown that cells adhere poorly to chitosan surfaces (Huang et al., 2011), but readily to chitin surfaces (Noh et al., 2006). We hypothesized that combining fibrillated chitosan and chitin nanofibers could simultaneously resolve the competing requirements of adhesion and aggregation control in suspension culture. A commercially available scaffold composed of fibrillated chitosan and chitin nanofibers, Cellhesion™-MS, has previously been shown to support MSC proliferation in static suspension culture by forming cell–scaffold aggregates without excessive aggregation (Kida et al., 2022; Zhu et al., 2024). However, these reports have been limited to static systems. For scale-up applications, translation to dynamic suspension culture is essential. In the present study, we focused on the behavior of the fibrillated chitosan and chitin nanofibers that constitute Cellhesion™-MS and utilized their functional properties as the foundation for designing a scalable dynamic suspension culture system for MSCs.

## 2 Materials and methods

### 2.1 Scaffolds preparation

Dispersions of fibrillated chitosan nanofibers (1% w/v) and fibrillated chitin nanofibers coated with vitronectin (1% w/v), which together comprise Cellhesion^TM^-MS (Kida et al., 2022; Zhu et al., 2024) (containing fibrillated chitosan and chitin nanofibers dispersions in a volume ratio of 80:20 (v/v)), were obtained from Nissan Chemical Corporation (Tokyo, Japan). Fluorescent labeling of scaffolds was performed as follows. Stock solutions of 2 mg/mL Fluorescein-4-isothiocyanate (FITC) (F007; DOJINDO) and 0.25 mg/mL California Red™ SE (473; AAT Bioquest) were prepared by dissolving each dye in dimethyl sulfoxide (DMSO) (031-24051; FUJIFILM Wako), sterilized using a 0.22 μm syringe filter, and added to the nanofiber dispersions to achieve final concentrations of 20 μg/mL FITC or 5 μg/mL California Red™ SE, followed by immediate mixing. After incubation for 2 h at room temperature in the dark, the supernatant was removed by centrifugation at 2,000 × *g* for 5 min, and the nanofibers were washed three times with distilled water.

### 2.2 Scanning electron microscopy (SEM)

Fibrillated chitosan and chitin nanofibers were suspended in 99.5% ethanol (057-00456; FUJIFILM Wako) for 2 h for dehydration, washed with t-butyl alcohol (028-03386; FUJIFILM Wako), applied to a glass plate, and lyophilized. After Pt sputtering deposition was performed, the specimens were subjected to SEM observation (JSM-7400F; JEOL). The accelerating voltage of the electron beam was 1 kV.

### 2.3 Scaffold size distribution analysis

Scaffolds were added to Mesenchymal Stem Cell Growth Medium 2 (C-28009; PromoCell) at final concentrations of 0.04% (w/v) fibrillated chitosan nanofibers or 0.01% (w/v) fibrillated chitin nanofibers, and transferred into 30-mL bioreactors (working volume: 30 mL, inner diameter: 36 mm, liquid height: 29 mm, BWV-S03A; ABLE) equipped with a delta-shaped paddle impeller. Suspensions were stirred on a magnetic stirrer (BWS-S03N0S-6C; ABLE) at 50 rpm in a humidified incubator at 37 °C, 5% CO_2_. At *t* = 24 h, before and after pipetting, samples were transferred to a glass cell and analyzed by laser diffraction particle size analysis using an LA-960 (HORIBA). Pipetting was performed using a 10-mL serological pipette (2-4131-14; AS ONE) at a flow rate of 3.8 ± 0.3 mL/s.

### 2.4 hMSC culture

Human adipose tissue-derived mesenchymal stem/stromal cells (MSCs) (0111201, Lot No. A19I22101, Male, 21 years old, *N*p = 2; CellSource) were used in this study. Cells were thawed from a purchased cryovial and seeded in growth medium at a viable cell density of 4 × 10^3^ cells/cm^2^ in T-75 flasks (430641U; Corning) and cultured in a humidified incubator at 37 °C, 5% CO_2_. After 3 days, cells were washed with D-PBS(−) (045-29795; FUJIFILM Wako) and detached by incubation with TrypLE™ Select Enzyme (12563011; Thermo Fisher) for 5 min at 37 °C. The cell suspension was centrifuged at 300 *g* for 3 min, and the pellet was resuspended in cryopreservation medium (STEM-CELLBANKER GMP grade, 11924; Zenogen Pharma) and aliquoted into cryovials. Cells were frozen at −80 °C overnight using a Corning CoolCell container (432002; Corning) and subsequently stored in the vapor phase of liquid nitrogen as a working cell stock. The stock cells were later expanded following the same procedure, and cells at passage number *N*p = 3 or 4 were used for experiments. Viable cell numbers were determined by the trypan blue exclusion method using 0.4% trypan blue solution (15250061; Thermo Fisher) and an automated cell counter (TC20; Bio-Rad).

### 2.5 Static suspension culture

Cells were seeded at a density of 1.5 × 10^4^ cells/mL in a 24-well low-attachment flat-bottom plate (MS-90240; Sumitomo Bakelite) under the following conditions: without scaffolds (w/o scaffolds) or with 0.05% (w/v) scaffolds containing chitosan:chitin ratios of 100:0 (Chitosan only), 80:20 (Chitosan/Chitin (80:20)), 50:50 (Chitosan/Chitin (50:50)), 20:80 (Chitosan/Chitin (20:80)), and 0:100 (Chitin only). Half-volume medium changes were performed once each at *t* = 48 h and 72 h, and three times at *t* = 96 h and every 24 h thereafter, for a total culture period of 168 h in a humidified incubator at 37 °C, 5% CO_2_. At *t* = 24, 96, and 168 h, cell density was measured, and the apparent specific growth rate (*μ* ^app^) was calculated according to Equation 1, where *t*_1_ and *t*_2_ represent the culture times (h) of interest, and *X*_*t*1_ and *X*_*t*2_ are the measured cell densities (cells/mL) at these time points.

$$\mu^{\text{app}}=\frac{\ln\left(X_{t_{2}}/X_{t_{1}}\right)}{t_{2}-t_{1}}$$ (1)

### 2.6 Aggregate dissociation and cell counting

Aggregate dissociation was performed by enzymatic treatment using a mixture of 0.14 mg/mL Liberase MNP-S (06297790001; Roche CustomBiotech) and 10X TrypLE™ Select Enzyme (A1217701; Thermo Fisher) at 37 °C for 20 min, with pipetting every 10 min, followed by resuspension in fresh medium. Cell density was determined by direct counting of viable cells using a hemocytometer and the trypan blue exclusion method.

### 2.7 Fluorescence time-lapse microscopy

Cells were stained with 10 μM CellTracker™ Green (C2925; Thermo Fisher) and seeded at 1.5 × 10^4^ cells/mL into low-attachment 35 mm dishes (80136; ibidi) containing 0.25% (w/v) scaffolds composed of fluorescently labeled chitosan only, fluorescently labeled chitin only, or a mixture of 20% fluorescently labeled chitin and 80% unlabeled chitosan. Cells were cultured for 48 h at 37 °C in a humidified atmosphere with 5% CO_2_. Time-lapse imaging was performed using an FV10i laser scanning microscope (Olympus), acquiring images every 30 min.

### 2.8 30-mL scale dynamic suspension culture for comparison of agitation

Cells were seeded at a density of 1.5 × 10^4^ cells/mL into 30-mL bioreactors (working volume: 30 mL) containing 0.05% (w/v) scaffolds with a chitosan:chitin ratio of 80:20 (Chitosan/Chitin (80:20)) and agitated at 25, 50, 80, or 100 rpm on a magnetic stirrer in a humidified incubator at 37 °C, 5% CO_2_. Cell densities were measured at *t* = 24, 48, 72, and 96 h. The specific growth rate (*μ*) was calculated as the slope of a linear regression of ln-transformed cell densities versus culture time over the exponential growth phase. The projected area of aggregates was determined from fluorescence images at *t* = 96 h.

### 2.9 30-mL scale dynamic suspension culture for comparison of nanofibers

Cells were seeded at a density of 1.5 × 10^4^ cells/mL into 30-mL bioreactors (working volume: 30 mL) under the following conditions: without scaffolds (w/o scaffolds), with 0.01% (w/v) chitin, or with 0.05% (w/v) scaffolds containing a chitosan:chitin ratio of 80:20 (Chitosan/Chitin (80:20)). Cultures were agitated at 50 rpm on a magnetic stirrer in a humidified incubator at 37 °C, 5% CO_2_. Half-volume medium changes were performed at *t* = 48 h and 72 h after seeding, and 80% medium changes at *t* = 96 and 108 h, followed by culture for 120 h. Medium changes were performed after 10 min sedimentation. Cell densities were measured at *t* = 24, 72, and 120 h, and the specific growth rate (*μ*) was calculated from these values. The projected aggregate area and number were determined from bright-field images at *t* = 120 h.

### 2.10 Live/dead staining analysis

Live/Dead staining was performed as follows. Sampled cells were rinsed with D-PBS(−) and stained with 1.0 μg/mL Calcein-AM (C326; DOJINDO) and 2.0 μg/mL Propidium iodide (PI) (P378; DOJINDO) in a multi-well plate for 15 min at 37 °C. Images were acquired using an EVOS FL Auto (Thermo Fisher).

### 2.11 Aggregate size distribution analysis

The projected area of aggregates was measured as follows. Whole-well fluorescence images of Live/Dead-stained aggregates were acquired using a Cell3iMager duos (SCREEN) or an IN Cell Analyzer 2000 (GE Healthcare). The projected areas of live-cell aggregates were measured using ImageJ (version 1.54p; National Institutes of Health, United States), and aggregates larger than 0.3 × 10^5^ μm^2^ were included in the analysis. Alternatively, whole-well bright-field images were obtained using a Cell3iMager duos, and image processing was performed using original codes which were adapted from the method reported by Hirono et al. (2024), executed in Python (version 3.9.13) using the NumPy (version 1.20.0) and OpenCV (version 4.4.0) libraries. The image-processing workflow consisted of: (1) Background flattening, (2) Contrast enhancement, (3) Binarization, (4) Object removal, (5) Dilation, (6) Erosion, (7) Dilation, (8) Masking, (9) Hole filling, and (10) Frame overlap removal. Relevant parameters for each step are described in the Supplementary Material. From each processed image, the projected area of aggregates was measured, and aggregates exceeding 0.3 × 10^5^ μm^2^ were analyzed. The aggregate number was calculated based on the sampling volume and the detected object count in the well.

### 2.12 Preparation and staining of frozen sections

Cells were seeded at a density of 1.5 × 10^4^ cells/mL into 30-mL bioreactors (working volume: 30 mL) under the following conditions: with 0.01% (w/v) FITC-labelled chitin only, or with 0.01% (w/v) FITC-labelled chitin and 0.04% (w/v) California Red-labelled chitosan (Chitosan/Chitin (80:20)). Cultures were agitated at 50 rpm on a magnetic stirrer in a humidified incubator at 37 °C, 5% CO_2_. Half-volume medium changes were performed at *t* = 48 h and 72 h, and 80% medium changes at *t* = 96 and 108 h, followed by culture for 120 h. Medium changes were performed after 10 min sedimentation. Aggregates were harvested at *t* = 72 and 120 h, rinsed with D-PBS(−), fixed with 4% paraformaldehyde (163-20145; FUJIFILM Wako) for 15 min at room temperature, rinsed again, and dehydrated overnight in 30% (w/v) sucrose (196-00015; FUJIFILM Wako). Aggregates were embedded in optimal cutting temperature (O.C.T.) compound (3801480; Leica Microsystems) and frozen using a hexane/isopentane mixture at −100 °C. Specimens were prepared by producing 10 µm thick slices of the aggregates using a cryostat (Leica CM3050 S; Leica). The slices were thaw-mounted onto adhesive glass slides (S7445; Matsunami Glass), dried for 30 min in the dark, rinsed with D-PBS(−), and permeabilized with 0.5% Triton X-100 (T8787-100ML; Sigma) for 5 min. After rinsing, specimens were incubated with a 100 ng/mL 4′,6-diamidino-2-phenylindole (DAPI) (D1306; Thermo Fisher) solution for 20 min to stain nuclei, rinsed again, and mounted with SlowFade mountant (S36936; Thermo Fisher) under a cover glass. Observation was performed using confocal laser scanning microscopy (FLUOVIEW FV1200; Olympus) with a ×40 objective lens.

### 2.13 1-L scale dynamic suspension culture

A 1-L glass bioreactor (working volume: 1 L, inner diameter: 114 mm, liquid height: 98 mm, BCP-02NP4; ABLE) equipped with a delta-shaped paddle impeller, dissolved oxygen (DO) sensor, and pH sensor was sterilized by autoclaving at 121 °C for 20 min. Growth medium containing 0.05% (w/v) Cellhesion^TM^-MS (Chitosan/Chitin (80:20)) was added using a peristaltic pump, and cells were seeded at a density of 1.5 × 10^4^ cells/mL. Cultures were maintained at 37 °C on a bioreactor control platform (BCP; ABLE) at 15 rpm. Air was supplied to the liquid surface at 80 ccm, and pH was maintained between 7.45 and 7.50 by intermittent CO_2_ supply. Oxygen was supplied to maintain DO at the initial level at 50 ccm by intermittent supply. Half-volume medium changes were performed at *t* = 48 h and 72 h, and 80% medium changes every 12 h from *t* = 96 h–132 h, followed by culture until 144 h. Medium changes were performed by pumping after 10 min sedimentation. The projected area of aggregates was measured from bright-field images at *t* = 120 h. Cell densities were determined at *t* = 24, 72, 120, and 144 h.

### 2.14 Flow cytometry

After obtaining single-cell suspensions at *t* = 144 h, cells were separated from nanofiber scaffolds using a 65 μm inline cell strainer (SCM200B; Nissan Chemical). Cells were washed with D-PBS(−) containing 2% (v/v) fetal bovine serum (FBS) (175012; NICHIREI BIOSCIENCES) and divided into three tubes. Each tube was incubated with conjugated antibodies for 30 min in the dark. Flow cytometry was performed using a BD LSRFortessa™ X-20 (BD), and data were analyzed using FlowJo software (version 10.6.1; BD). All antibodies were purchased from BD, and dilution ratios were according to the manufacturer’s instructions. Tube 1 (isotype control): FITC Mouse IgG1, κ Isotype Control (554679), PE Mouse IgG1, κ Isotype Control (555749), BV421 Mouse IgG1, k Isotype Control (562438), APC Mouse IgG1, κ Isotype Control (555751). Tube 2 (specific antibodies): FITC Mouse anti-Human CD11b (562793), PE Mouse Anti-Human CD34 (555822), BV421 Mouse Anti-Human CD73 (562430), APC Mouse Anti-Human CD90 (559869). Tube 3 (specific antibody): PE Mouse anti-Human CD105 (560839).

### 2.15 Trilineage mesenchymal differentiation

After obtaining single-cell suspensions at *t* = 144 h, cells were cryopreserved, then thawed and seeded for each differentiation. Adipogenic differentiation was performed using Adipogenic Differentiation Medium (C-28016; PromoCell) following the manufacturer’s instructions. Briefly, cells were seeded with growth medium in 12-well plates (3513; Corning) coated with fibronectin (F0895-1 MG; Merck) and cultured until 80% confluence. The medium was then replaced with differentiation medium and cultured for 12 days with medium changes every third day. After fixation with 4% paraformaldehyde for 15 min, cells were stained with Oil-Red O solution (BMK-R007; Bio Future Technologies) according to the manufacturer’s instructions. Osteogenic differentiation was conducted using Osteogenic Differentiation Medium (C-28013; PromoCell) as above. Cells were seeded in fibronectin-coated 12-well plates, cultured until 100% confluence, and then treated with differentiation medium for 12 days with medium changes every third day. After fixation, cells were stained with Alizarin Red S solution (BMK-R009; Bio Future Technologies). Chondrogenic differentiation was performed using Chondrogenic Differentiation Medium (C-28012; PromoCell). Briefly, 2 × 10^5^ cells were seeded in low-glucose DMEM (041-29775; FUJIFILM Wako) containing 10% (v/v) FBS in 96-well U-bottom plates (MS-9096U; Sumitomo Bakelite). After 2 days of culture, the medium was replaced with differentiation medium and cultured for 19 days with medium changes every third day. After fixation, cells were stained with Alcian Blue solution (BMK-R011; Bio Future Technologies). Images of stained cells were acquired using an inverted microscope (IX73; Olympus).

### 2.16 5-L scale dynamic suspension culture

A 5-L glass bioreactor (working volume: 5 L, inner diameter: 226 mm, liquid height: 125 mm, custom made; ABLE) equipped with a delta-shaped paddle impeller, DO sensor, and pH sensor was sterilized by autoclaving at 121 °C for 20 min. Growth medium containing 0.05% (w/v) Cellhesion^TM^-MS (Chitosan/Chitin (80:20)) was added via a peristaltic pump, and cells were seeded at 1.0 × 10^4^ cells/mL. Cultures were maintained at 37 °C on a BCP at 18 rpm. Air was supplied to the liquid surface at 600 ccm, and pH was maintained between 7.45 and 7.50 by intermittent CO_2_ supply. Oxygen was supplied to maintain DO at the initial level (200 ccm). Half-volume medium changes were performed at *t* = 72 h and 96 h, and a 70% medium change at *t* = 120 h, followed by culture until *t* = 144 h. Medium changes were performed by pumping after 10 min sedimentation. The projected area of each aggregate was measured from fluorescence images at *t* = 120 h, and cell densities were determined at *t* = 96, 120, and 144 h.

### 2.17 Statistical analysis

Statistical significance was determined using Tukey’s test or Student’s *t*-test in R (version 4.4.2). Differences with *p* < 0.05 and *p* < 0.01 were considered statistically significant.

## 3 Results

### 3.1 Properties of fibrillated chitosan and chitin nanofibers

**FIGURE 1:**
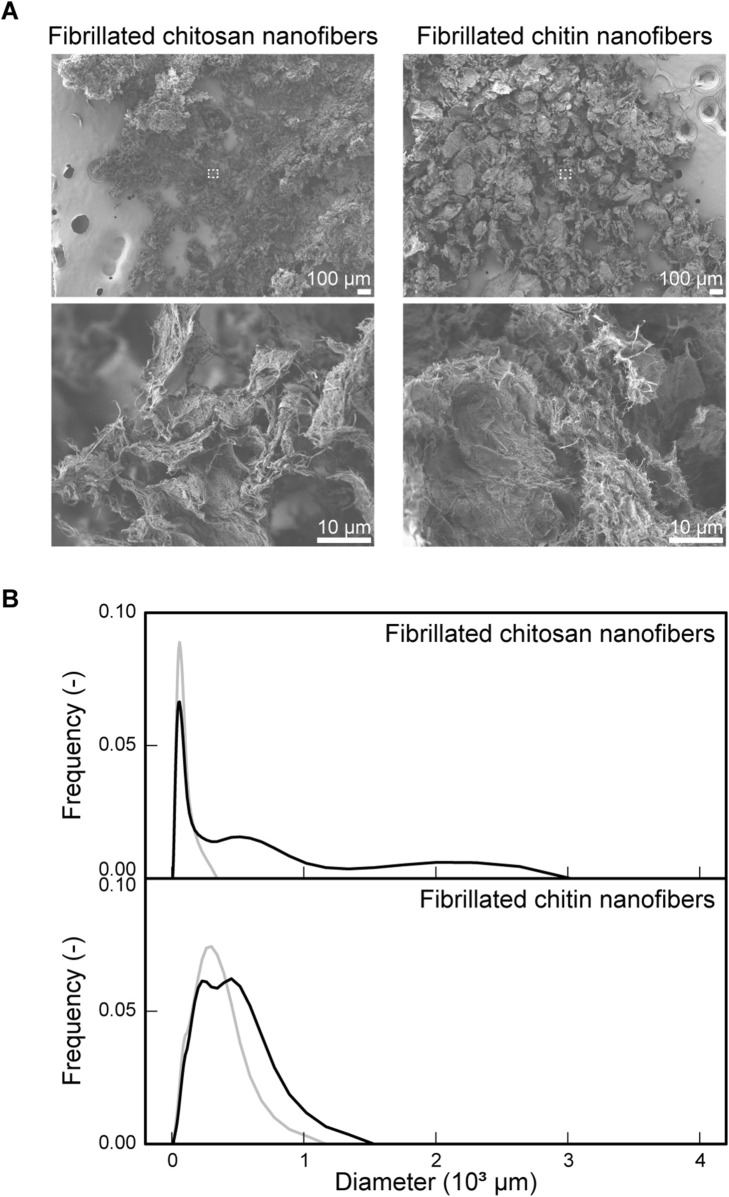
Properties of fibrillated chitosan and chitin nanofibers. **(A)** Representative SEM images of fibrillated chitosan and chitin nanofibers at × 50 (top, scale bar: 100 μm) and × 2,000 (bottom, scale bar: 10 μm) magnification. The dotted region in the × 50 image corresponds to the field observed at ×2,000 magnification. **(B)** Particle-size distributions of fibrillated chitosan and chitin nanofibers after 24 h of suspension in a 30-mL bioreactor, shown before pipetting (black) and after pipetting (gray).

To investigate the structural properties of fibrillated chitosan and chitin nanofibers, SEM observations were conducted. Both fibrillated chitosan and chitin nanofibers exhibited irregular particle shapes, although the shape of chitin nanofibers appeared more well defined than that of the chitosan nanofibers (Figure 1A). Higher-magnification views revealed that both scaffolds possessed randomly oriented fibrous surface structures, in which nanometer-scale fibers were entangled with one another (Figure 1A). Local variations in fiber density contributed to the formation of porous structures, with chitosan nanofibers exhibiting more branched features characterized by fibrillated structures than chitin nanofibers. To investigate the dynamics of these nanofibers in suspension, particle size distributions were measured. Chitosan nanofibers exhibited three peaks after 24 h of suspension: a sharp major peak at a smaller diameter and two broader minor peaks (Figure 1B). Following pipetting, the minor peaks disappeared, and the distribution shifted to a single, smaller peak. Chitin nanofibers showed a broad peak after 24 h of suspension, the shoulder of which shifted toward a narrower, smaller peak after pipetting, exhibiting behavior similar to that of chitosan nanofibers (Figure 1B). These results indicate that the scaffolds spontaneously formed weak agglomerations that were readily dispersed by pipetting.

### 3.2 Static suspension culture with various nanofiber conditions

**FIGURE 2:**
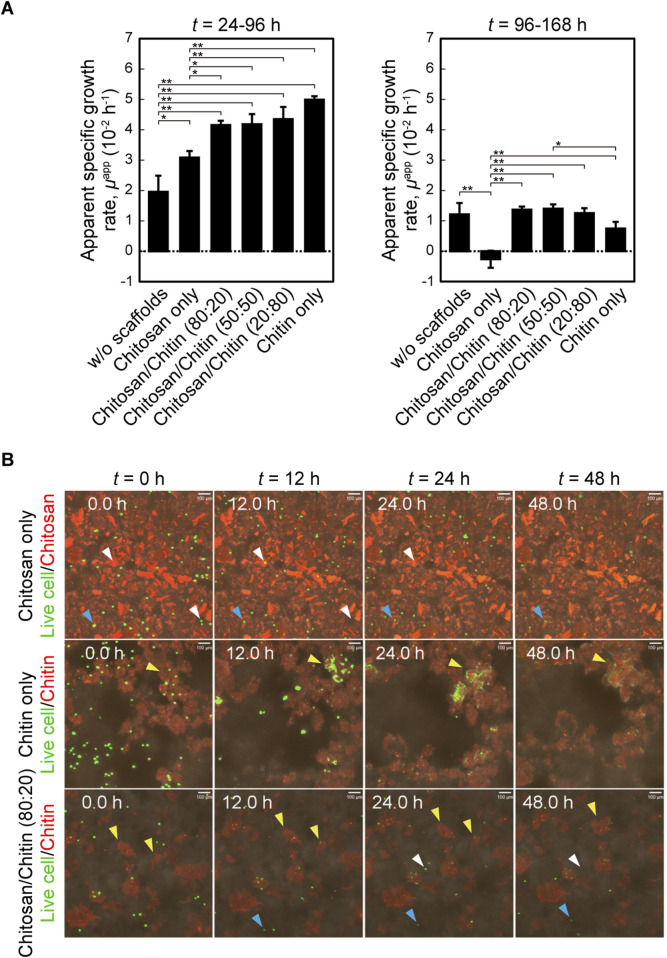
Static suspension culture under various nanofiber conditions. **(A)** Apparent specific growth rate (*μ* ^app^) during 24–96 h (left) and 96–168 h (right). Data are mean ± s.d. *: *p* < 0.05, **: *p* < 0.01 (Tukey’s test, *n* = 3 per culture). **(B)** Snapshot images from time-lapse observation of cells cultured with California Red-labelled chitosan and/or chitin nanofibers (red) and CellTracker-labelled cells (green) under static conditions. Arrows indicate a moved cell (white), a non-extending cell remaining on the scaffold (blue), and an extending cell (yellow).

To investigate the growth properties of MSCs cultured with fibrillated nanofibers, cells were cultured under static suspension conditions with varying chitosan/chitin scaffold ratios. Under scaffold-free and chitosan-only conditions, the apparent specific growth rates (*μ* ^app^) were lower than those observed under chitin-containing conditions during the early phase (24–96 h) (Figure 2A). In chitin-containing conditions, a higher chitin ratio was associated with a slight increase in *μ* ^app^ during the early phase, whereas a decline was observed in the late phase (96–168 h). Time-lapse observations revealed that chitin nanofibers gradually entangled with neighboring scaffolds to form cell–scaffold aggregates, whereas higher chitosan nanofiber content resulted in more dispersed and smaller aggregates (Supplementary Video S1). Under the chitosan/chitin (80:20) condition, the sizes of the cell–scaffold aggregates were smaller than those in other chitin-containing conditions, and cells continued proliferating until *t* = 168 h; thus, subsequent analyses were conducted under this condition. Cell behavior on each scaffold was observed using fluorescent labeling. Under the chitosan-only condition, the number of cells in the observed field decreased over time (white arrow), although some cells remained on the nanofiber scaffolds (blue arrow) (Supplementary Video S2; Figure 2B). Cells contacting chitosan nanofiber scaffolds exhibited spherical morphologies without extension (Supplementary Video S3). In contrast, cells in chitin-only cultures initially exhibited spherical shapes but extended along the scaffold surfaces within 24 h (Supplementary Video S3). Subsequently, they migrated and divided, incorporating surrounding scaffolds and forming cell–scaffold aggregates (yellow arrow), while chitin nanofibers gradually agglomerated. In the chitosan/chitin (80:20) condition, in which chitosan nanofibers were unlabeled and chitin nanofibers were fluorescently labeled, a combination of these behaviors was observed. Notably, partial cell–scaffold aggregation occurred, but the chitin nanofibers remained more dispersed than in the chitin-only condition. These observations suggest that chitin nanofibers provided scaffolds that supported cell adhesion, division, and migration, leading to the formation of cell–scaffold aggregates through their own agglomeration. In contrast, chitosan nanofibers lacked adhesiveness and prevented such agglomeration.

### 3.3 Suspension culture using mixed chitosan and chitin nanofiber scaffolds

**FIGURE 3:**
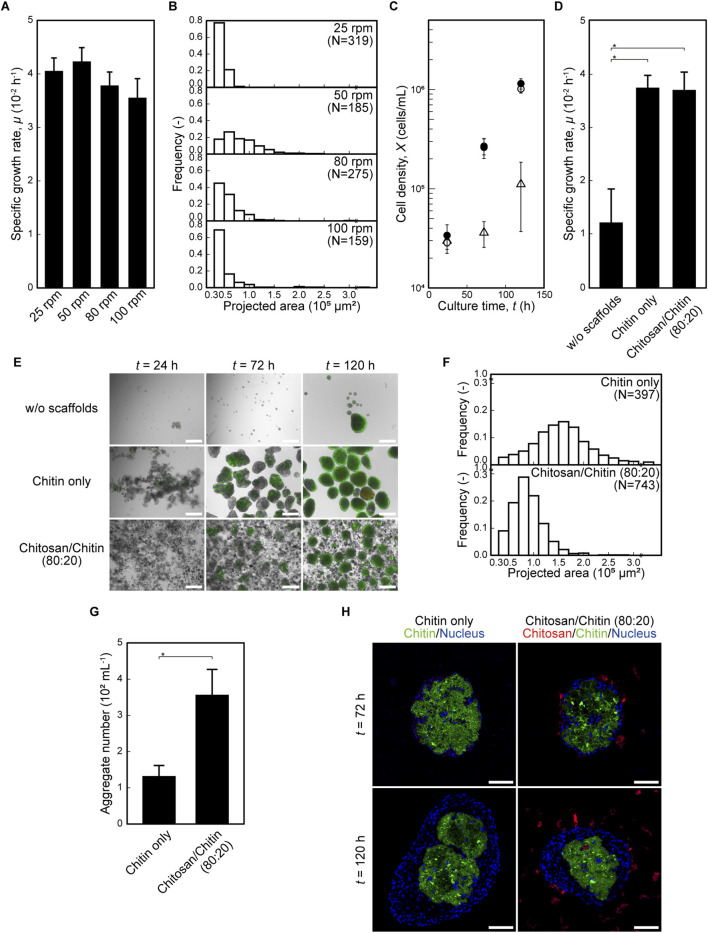
Dynamic suspension culture using mixed of chitosan and chitin nanofiber scaffolds. Comparison of agitation conditions in the presence of chitosan/chitin (80:20) using a 30-mL bioreactor. **(A)** Specific growth rate (*μ*) during 24–96 h. Data are mean ± s.d.; no significant difference was detected (Tukey’s test, *n* = 3 per culture). **(B)** Distribution of aggregate projected area at *t* = 96 h. Comparison of scaffold conditions at 50 rpm in a 30-mL bioreactor. **(C)** Growth profiles under without scaffolds (open triangle, △), chitin only (open circle, ○), and chitosan/chitin (80:20) (closed circle, ●). Data are mean ± s.d. (*n* = 5 per culture). **(D)** Specific growth rate (*μ*) during 24–120 h. Data are mean ± s.d. *: *p* < 0.01 (Tukey’s test, *n* = 5 per culture). **(E)** Representative Live/Dead staining images: live cells (Calcein-AM, green) and dead cells (Propidium iodide, PI, red). Scale bar: 500 μm. **(F)** Distribution of aggregate projected area over 0.3 × 10^5^ μm^2^ at *t* = 120 h. **(G)** Aggregate number over 0.3 × 10^5^ μm^2^ at *t* = 120 h. Data are mean ± s.d. *: *p* < 0.01 (Student’s t-test, *n* = 5 per culture). **(H)** Confocal fluorescence images of aggregate sections cultured with FITC-labelled chitin (green) only or with California Red-labelled chitosan (red) at *t* = 72 and 120 h using a 30-mL bioreactor at 50 rpm. Blue shows nuclei (DAPI). Scale bar: 100 μm.

To evaluate the potential of the nanofiber mixture as scaffolds, the mixture was applied to a dynamic suspension culture system. First, the effects of agitation rate on cell–scaffold aggregation and subsequent cell proliferation were evaluated at 25, 50, 80, and 100 rpm. MSCs proliferated under all conditions, showing no significant difference in specific growth rate (*μ*), although the highest *μ* was observed at 50 rpm (Figure 3A). The sizes of cell–scaffold aggregates at *t* = 96 h were larger at 50 rpm than at 25 rpm and shifted toward smaller sizes as the agitation rate increased to 80 and 100 rpm (Figure 3B). Under the chitosan/chitin (80:20) condition, gentle agitation that formed moderately sized cell–scaffold aggregates—while avoiding excessive shear at higher agitation rates—supported efficient cell proliferation. Accordingly, 50 rpm was selected for subsequent 30-mL bioreactor cultures, which were compared with those using chitin-only scaffolds. Under both scaffold conditions, cell proliferation was comparable, and *μ* values were higher than in the scaffold-free condition (Figures 3C,D). In the chitin-only condition, cells extended at *t* = 24 h and subsequently formed cell–scaffold aggregates by *t* = 72 h. The aggregates further increased in size by *t* = 120 h, and PI-positive dead cells were observed in the centers of these aggregates (Figure 3E). In the chitosan/chitin (80:20) condition, cells exhibited similar extension and aggregate formation; however, the aggregates appeared smaller, and PI-positive dead cells were less frequently observed than in the chitin-only condition (Figure 3E). Aggregate size distributions at *t* = 120 h under the chitin-only condition ranged broadly from 0.3 to 3 × 10^5^ μm^2^, with a peak between 1.5 and 1.7 × 10^5^ μm^2^, including populations exceeding 3 × 10^5^ μm^2^. By contrast, the chitosan/chitin (80:20) mixture showed no aggregates larger than 3 × 10^5^ μm^2^ and exhibited a narrower distribution peaking at 0.7–0.9 × 10^5^ μm^2^ (Figure 3F). Additionally, the number of aggregates was significantly higher under the chitosan/chitin (80:20) condition than under the chitin-only condition (Figure 3G). Histological analysis of aggregate sections revealed that cells were distributed between and around the chitin nanofiber scaffolds within the aggregates, whereas chitosan nanofiber scaffolds were predominantly located at the periphery of the aggregates, adjacent to the outer cell surfaces (Figure 3H). Taken together, these results indicate that under dynamic culture, chitosan and chitin nanofiber scaffolds exhibited behaviors similar to those observed under static conditions. Chitin nanofibers supported cell extension and the formation of cell–scaffold aggregates that enhanced proliferation, whereas chitosan nanofibers, located between aggregates, physically inhibited their contact and thus prevented coalescence, resulting in smaller cell–scaffold aggregates with fewer dead cells.

### 3.4 Scaling-up of suspension culture using fibrillated nanofiber scaffolds

**FIGURE 4:**
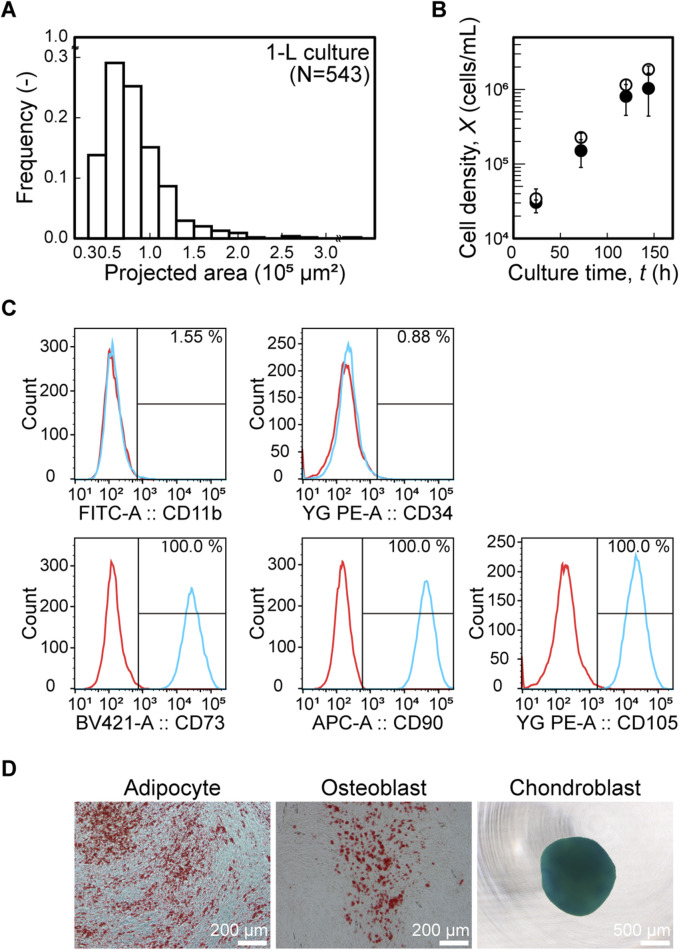
Application of nanofiber scaffolds to 1-L scale culture. **(A)** Distribution of aggregate projected area over 0.3 × 10^5^ μm^2^ at *t* = 120 h in 1-L culture. **(B)** Growth profiles in 30-mL (open circle, ○) and 1-L (closed circle, ●) bioreactors. Data are mean ± s.d. (*n* = 3 per culture). **(C)** Flow-cytometric analysis of MSC surface-marker expression at *t* = 144 h in 1-L culture (isotype control, red; specific antibody, blue). **(D)** Differentiation potential after 1-L culture toward adipocyte and osteoblasts (Scale bar: 200 μm) and chondroblast (Scale bar: 500 μm).

**FIGURE 5:**
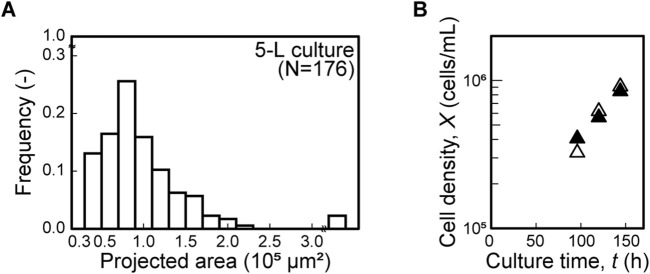
Application of nanofiber scaffolds to 5-L scale culture. **(A)** Distribution of aggregate projected area over 0.3 × 10^5^ μm^2^ at *t* = 120 h in 5-L culture. **(B)** Growth profiles in 30-mL (open triangle, △) and 5-L (closed triangle, ▲) bioreactors. Data represent a single experiment (*n* = 1 per culture).

To evaluate the scalability of the culture system employing fibrillated chitosan and chitin nanofiber scaffolds, dynamic cultures were first performed at the 1-L scale. To reproduce the cellular microenvironment, agitation conditions were scaled up based on the size distribution of the cell–scaffold aggregates. In the 1-L stirred bioreactor, continuous gentle agitation at 15 rpm from inoculation enabled aggregate formation (Supplementary Video S4), resulting in a size distribution similar to that observed in the 30-mL culture (Figure 4A). Under this condition, the time course of viable cell density in the 1-L culture followed a trend similar to that of the 30-mL culture (Figure 4B). At *t* = 144 h, the viable cell density reached (1.03 ± 0.59) × 10^6^ cells/mL in the 1-L culture, which was comparable to (1.85 ± 0.20) × 10^6^ cells/mL in the 30-mL culture. MSCs cultured at the 1-L scale expressed typical MSC surface markers, being positive for CD73, CD90, and CD105 and negative for CD11b and CD34 (Figure 4C). They also demonstrated trilineage differentiation into adipocytes, osteoblasts, and chondroblasts (Figure 4D), with negative staining confirmed at the initiation of differentiation (Supplementary Figure S1). To further assess the scalability of the fibrillated chitosan and chitin nanofiber scaffolds, this system was applied to a 5-L culture a single run (N = 1), in which the bioreactor geometry differed from that of the 30-mL and 1-L systems. At this scale, a similar size distribution of cell–scaffold aggregates was observed under continuous agitation at 18 rpm (Figure 5A), and the growth profile resembled that of the 30-mL bioreactor (Figure 5B). The total cell yield reached 4.23 × 10^9^ after 144 h of culture in a single 5-L bioreactor. Thus, in this nanofiber scaffold-based culture system, comparable cell growth environments were successfully reproduced across different scales, enabling scale-up to 5 L based on the cell–scaffold aggregate environment. Key parameters and performance metrics for all scales are summarized in Table 1.

**TABLE 1:** Summary of scale-up culture parameters and performance.

Parameters/Indicators	30-mL bioreactor	1-L bioreactor	5-L bioreactor
Vessel geometry			
Working volume (L)	3.0 × 10^−2^	1.0	5.0
Inner diameter (mm)	36	114	226
Liquid height (mm)	29	98	125
Operating conditions			
Agitation rate (rpm)	50	15	18
Seeding density (10^4^ cells/mL)	1.5	1.5	1.0
Performance metrics			
Specific growth rate, *μ* (10^−2^ h^-1^)	3.66 ± 0.28	3.27 ± 0.40	3.50
Peak viable cell density at *t* = 144 h (10^6^ cells/mL)	1.85 ± 0.20	1.03 ± 0.59	0.85
Total cell yield at *t* = 144 h (cells)	(5.56 ± 0.60) × 10^7^	(1.03 ± 0.59) × 10^9^	4.23 × 10^9^
Aggregate area range at *t* = 120 h	-	Similar to 30-mL	Similar to 30-mL

## 4 Discussion

**FIGURE 6:**
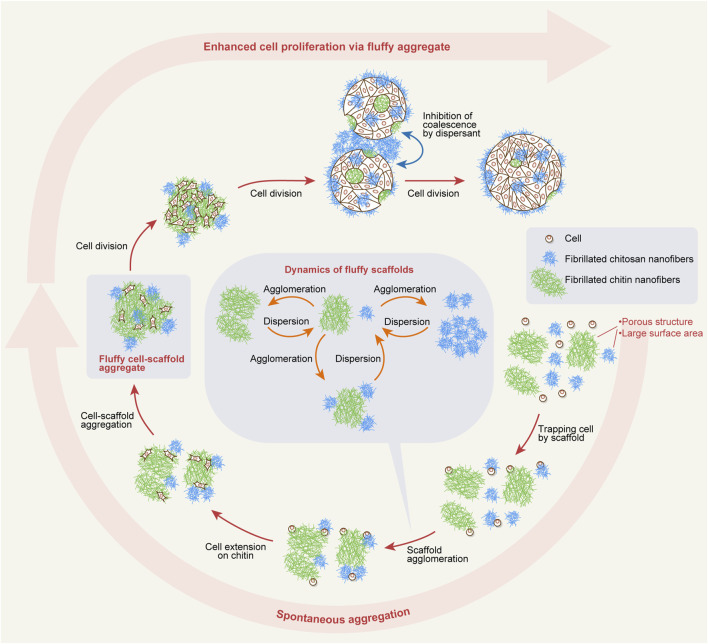
Schematic illustration of scaffold and cell behaviors in dynamic suspension culture.

This study demonstrates a scalable suspension culture system for MSCs based on a novel, biology-centric design principle. The unique properties of fibrillated chitosan and chitin nanofibers enabled the creation of a self-organizing cellular microenvironment that could be consistently reproduced across different culture scales. Our proposed mechanism for this process is illustrated in Figure 6. First, the scaffolds exhibit fiber entanglement, which induces agglomeration and enables them to trap dispersed cells. This trapping occurs both intrinsically and during agglomeration, after which cell extension occurs, especially on chitin nanofibers. These entanglement and cell-extension events facilitate the formation of fluffy cell–scaffold aggregates that stabilize suspended cells for division. This spontaneous aggregation occurs over several hours. Within these aggregates, cell migration and division are promoted on chitin nanofibers located in the interior regions over several days. During this expansion phase, aggregates readily form larger ones through coalescence in the absence of chitosan nanofibers, whereas chitosan nanofibers localized at the aggregate periphery and in the bulk medium prevent coalescence.

The random network structure of nanofibers is known to promote physical entanglement owing to their intrinsic properties (Xu et al., 2024), which likely enables the scaffolds to trap cells before adhesion occurs. Chitosan surfaces are known to exhibit poor cell adhesiveness (Huang et al., 2011), whereas chitin surfaces have the potential to support cell adhesion (Noh et al., 2006). Consequently, cells adhered to chitin nanofibers and were located within the aggregate interior, while chitosan nanofibers were primarily positioned around the periphery or in the surrounding medium.

A structure in which cells are in the gaps of scaffolds resembles that of porous microcarriers. Compared with non-porous microcarriers, these structures provide a larger adhesive surface area, resulting in higher-density culture (Nilsson et al., 1986). On the other hand, cell aggregation within pores is difficult to control because it depends on cell migration and pore size during seeding (Yuan et al., 2014). In our culture system, cells and nanofiber scaffolds spontaneously formed fluffy aggregates under simple suspension conditions, allowing cells to proliferate between and on the scaffold surfaces within aggregates. These features provide a scalable culture platform that supports enzymatic dissociation and facilitates high-density culture, thereby addressing a major limitation of conventional porous microcarriers, in which cell harvesting is often difficult (Mizukami et al., 2018).

In dynamic suspension cultures, dispersed MSCs are vulnerable to shear-induced damage and anoikis (Frith et al., 2010), limiting growth (Sart et al., 2014). Thus, an initial static culture period is often employed to promote aggregate formation before initiating dynamic culture (Frith et al., 2010). Nevertheless, MSC aggregates may exhibit reduced growth because of compression (Sart et al., 2014) or necrosis at their cores caused by restricted mass transfer (Egger et al., 2018). To mitigate this, Hayashi et al. embedded gelatin microparticles within aggregates (Hayashi and Tabata, 2011), suggesting that relaxation of the aggregate structure can maintain mass transfer and reduce compression, thereby enhancing proliferation by forming a fluffy cell–scaffold aggregate structure. Given the limited proliferation typically observed in MSC aggregates, microcarriers are widely used in large-scale suspension cultures (Silva Couto et al., 2020; Tsai et al., 2020). As with aggregate formation, initial cell adhesion to microcarriers under dynamic conditions is challenging (Hewitt et al., 2011; Yuan et al., 2014; Moreira et al., 2020). Cell adhesion to a surface involves several sequential steps: physical contact between cells and scaffolds via electrostatic interactions; integrin-mediated binding to scaffold substrates; and formation of focal adhesions (Hong et al., 2006). Under dynamic conditions, physical contact is limited, and hydrodynamic forces generated by agitation can detach cells after adhesion (Forestell et al., 1992; Hewitt et al., 2011). Under static conditions, cells can readily encounter and adhere to microcarriers; however, static culture lacks control over cell and microcarrier settling, leading to non-adherent microcarriers or excessive aggregation. Consequently, intermittent agitation strategies—designed to minimize flow, promote cell–microcarrier encounters and adhesion, and maintain adequate dispersion—have been widely adopted (Yuan et al., 2014; Tsai and Pacak, 2021). To determine optimal operating conditions under these strategies, numerous combinations must be screened, involving variables such as microcarrier concentration, cell density, agitation duration and rate, resting time, number of intermittent cycles, and sedimentation time of cells and microcarriers. Yuan et al. screened several agitation rates during intermittent agitation in the seeding phase, which improved attachment but also induced aggregation at the 125-mL scale (Yuan et al., 2014), presumably due to poor mixing. This suggests that achieving both efficient cell adhesion and sufficient dispersion during seeding is challenging even at small scales. When scaled up, increased liquid depth prolongs the time required for cells to settle and adhere to microcarriers, implying that cell and microcarrier behaviors may vary among bioreactors of different sizes and become increasingly difficult to control. Therefore, it is essential to design highly scalable operating conditions based on a clear understanding of both the material properties and the process itself (Kino-oka et al., 2019). Our system overcomes these challenges by leveraging the unique physical properties of the fluffy scaffolds, such as higher apparent bulk density compared to polystyrene-based microcarriers (Supplementary Figure S2). Their fibrous and bulky structure containing many voids allows them to remain suspended under very gentle agitation, which fundamentally alters the process dynamics. This enables the use of continuous agitation from the point of inoculation, a simpler and more scalable alternative to intermittent protocols. Given the challenges associated with intermittent agitation, Ebrahimian et al. adopted continuous agitation as a more scalable alternative because of its reduced operational complexity (Ebrahimian et al., 2024). They focused on three variables—microcarrier concentration, cell density, and agitation rate—and applied a three-level design-of-experiments (DoE) approach, testing 21 of 3^3^ designed conditions to identify optimal operating parameters (Ebrahimian et al., 2024). Although its applicability remains limited by the need for high-shear agitation to disperse microcarriers, the nanofiber scaffolds used in this study, which exhibited fluffy behavior, remained dispersed under gentle agitation. This minimized shear stress while enabling cell adhesion and scaffold dispersion—two conditions traditionally at odds.

Excessive aggregation during the culture tends to occur under gentle agitation (Jossen et al., 2018; Zhang et al., 2023). In this study, non-adherent chitosan nanofiber scaffolds functioned as a dispersant, spacing aggregates during the expansion phase. This helped maintain the microenvironment of fluffy cell–scaffold aggregates regardless of culture scale. Under dynamic conditions, agitation intensity and the chitosan/chitin ratio are potential parameters for achieving the optimal size of the cell–scaffold aggregates. Although increasing agitation reduces scaffold agglomeration, it may also cause shear-induced cell damage, thereby limiting the maximum applicable agitation intensity. Given that the chitosan/chitin (80:20) condition achieved cell growth comparable to that of the chitin-only condition while resulting in fewer dead cells, a chitosan/chitin ratio of 80:20 appears to be sufficient under dynamic conditions.

In anchorage-dependent cell culture, scale-up requires designs that provide homogeneity of the cellular environment. Shear stress is a critical environmental property, and the just-suspended speed (*N*_js_)—the minimum agitation rate required to ensure that no microcarriers remain on the bottom of the vessel for more than one to two seconds—is sometimes used as a basal parameter for scale-up (Schirmaier et al., 2014; Lawson et al., 2017). While these strategies have shown success in certain scale-up scenarios (Schirmaier et al., 2014; Lawson et al., 2017), their applicability may be limited by differences in cell-attachment properties. Consequently, considerable effort is often needed to identify optimal strategies, such as sequential agitation conditions for the seeding and expansion phases (Lawson et al., 2017) or the use of additional devices optimized for seeding (Schirmaier et al., 2014). For our fluffy scaffolds, the strategy for determining agitation conditions is conceptually similar to that for microcarriers (Jossen et al., 2016), in which cell proliferation depends on agitation intensity, although the operational range is broader. Their fluffy behavior lowers *N*_js_, thereby reducing shear stress. Furthermore, scaffold agglomeration promotes the formation of cell–scaffold aggregates even under continuous agitation, thereby enabling a simple seeding operation and allowing cells to proliferate within aggregates while maintaining dispersion through their fluffy structure. Under these conditions, comparable specific growth rates from *t* = 0–120 h were achieved across scales: (3.66 ± 0.28) × 10^−2^ h^-1^ in 30-mL, (3.27 ± 0.40) × 10^−2^ h^-1^ in 1-L, and 3.50 × 10^−2^ h^-1^ in 5-L (N = 1) cultures, although the 5-L one remains preliminary. These findings suggest that the biology-centric, fluffy-environment-based scaling strategy, which encompasses both scaffold behavior and aggregate structure, simplifies conventional engineering parameters into a single aggregate-size criterion that captures complex hydrodynamic changes and provides a robust framework for developing scalable culture processes.

## 5 Conclusion

We designed a suspension culture system that enables scalable operation under continuous agitation by employing fluffy scaffolds composed of fibrillated chitosan and chitin nanofibers. These scaffolds support cell–scaffold aggregation during the seeding phase and maintain dispersion during the expansion phase. During seeding, fluffy cell–scaffold aggregation was promoted by the characteristics of the nanofibers, which trap dispersed cells, promote cell–scaffold aggregation, and enhance cell proliferation. During expansion, aggregates remained dispersed owing to the non-adhesive nature of chitosan nanofibers, which suppressed excessive aggregation. From a process perspective, the unique behavior of the fluffy scaffolds minimized shear stress and enabled consistent culture under continuous, gentle agitation. By adjusting agitation conditions based on cell–scaffold aggregate size, comparable culture efficiency was achieved across bioreactors of different scales, demonstrating both the high scalability of this culture system and its potential to adapt to vessel size during scale-up. This fluffy-environment-based scaling strategy using nanofiber scaffolds represents a significant advance in developing scalable culture processes for anchorage-dependent cells.

## Data Availability

The original contributions presented in the study are included in the article/Supplementary Material, further inquiries can be directed to the corresponding author.
