# Supplementary material for: A fluffy nanofiber scaffold-based microenvironment enables a simplified, biology-centric scale-up strategy for mesenchymal stem/stromal cell culture
Source: Front Bioeng Biotechnol. 2026 Apr 14;14:1808384. doi: 10.3389/fbioe.2026.1808384 (PMC13121900; doi:10.3389/fbioe.2026.1808384)
Supplement: Supplementary file 2 [file Supplementaryfile1.docx]

Supplementary Material

## Supplementary Figures


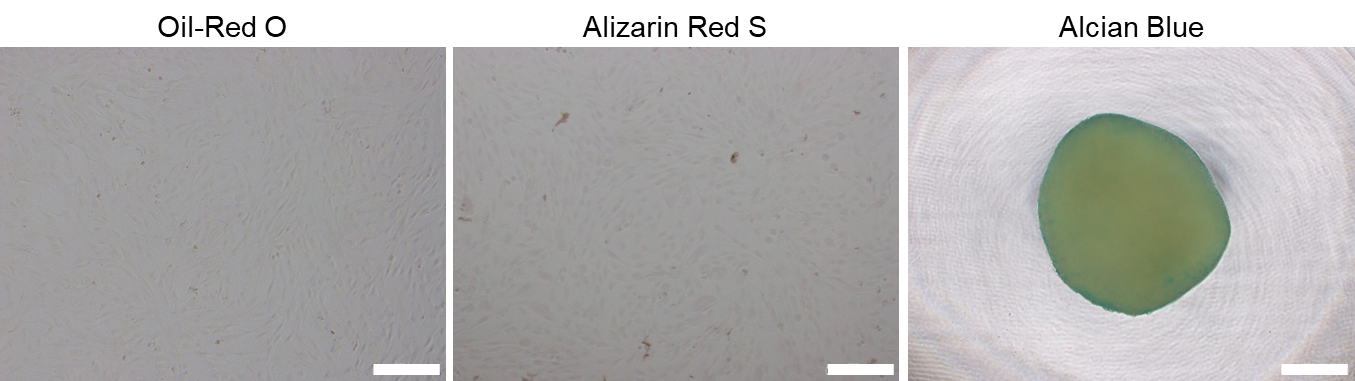


**Supplementary Figure 1.** Representative staining images at the initiation of differentiation (Day 0): Oil-Red O, Alizarin Red S (scale bar: 200 μm), and Alcian Blue (scale bar: 500 μm).


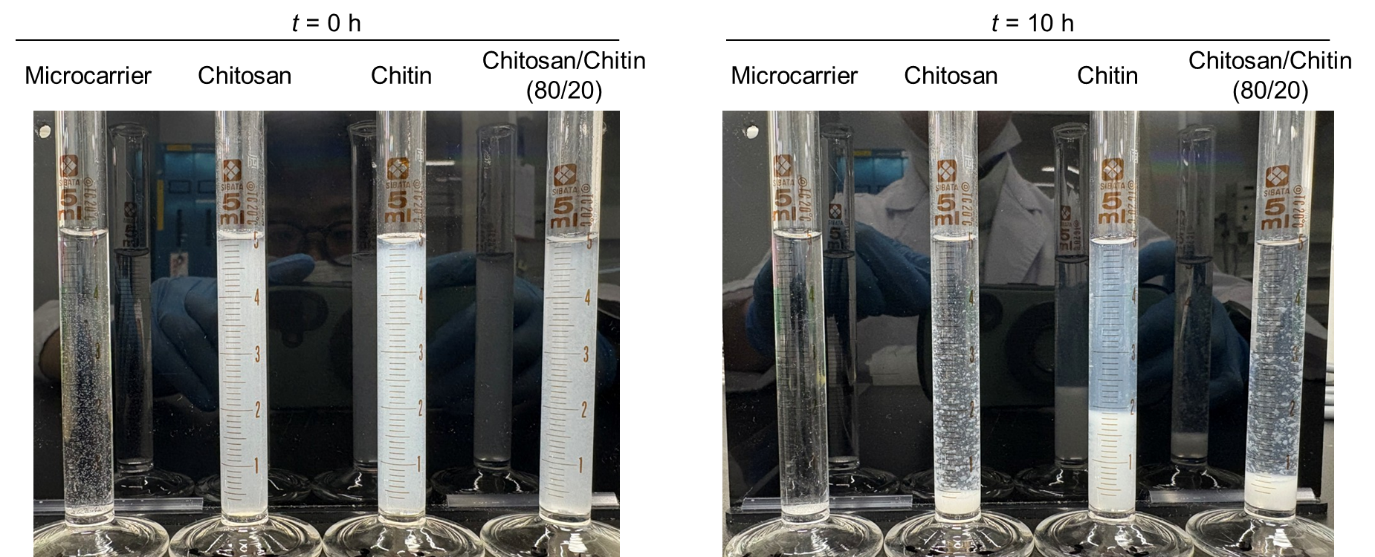


**Supplementary Figure 2.** Images showing the apparent bulk density at *t* = 0 h (left) and *t* = 10 h (right).

## Supplementary materials and methods

## Phase-contrast time-lapse observation

Cells were seeded at a density of 1.5 × 10^4^ cells/mL onto a low-attachment 96-well flat-bottom plate (3474; Corning) under the following conditions: without scaffolds (w/o scaffold), or with 0.05 % (w/v) scaffolds containing chitosan:chitin ratios of 100:0 (Chitosan only), 80:20 (Chitosan/Chitin (80:20)), 50:50 (Chitosan/Chitin (50:50)), 20:80 (Chitosan/Chitin (20:80)), and 0:100 (Chitin only). Half-volume medium changes were performed three times at *t* = 96 h after seeding and every 24 h thereafter until the total culture period reached 168 h at 37 °C in a humidified atmosphere containing 5 % CO_2_. Time-lapse observation was performed using a BioStudio-T (Nikon), with images acquired every 30 min.

## Stress fiber staining

Cells were seeded at a density of 1.5 × 10^4^ cells/mL into 30-mL bioreactors (working volume: 30 mL) containing either 0.04 % (w/v) California Red-labelled chitosan nanofibers or 0.01 % (w/v) FITC-labelled chitin nanofibers, and cultured under agitation at 50 rpm in a humidified incubator at 37 °C and 5 % CO_2_. At *t* = 24 h, cells were rinsed with D-PBS(-), fixed with 4 % paraformaldehyde for 15 min at room temperature, rinsed again, permeabilized with 0.5 % Triton X-100 for 5 min, and rinsed once more with D-PBS(-). Specimens were then stained with Phalloidin-iFluor™ 647 Conjugate (23127; AAT Bioquest) at a 1:1000 dilution for 30 min according to the manufacturer’s protocol. After rinsing with D-PBS(-), specimens were incubated with 100 ng/mL DAPI for 20 min to stain nuclei, rinsed again, and mounted on 35 mm glass-bottom dishes (D11130H; Matsunami Glass) with cover glasses. Observation was then performed using confocal laser scanning microscopy (FLUOVIEW FV1200; Olympus) equipped with a 60x objective lens.

## Image processing procedure

Image processing was performed based on the method reported by Hirono et al.(Hirono et al., 2024) with modifications.

(1) Background flatten: The original image was blurred with a 20 × 20 kernel (cv2.blur(original image, (20, 20))), and the background was flattened by dividing the original image by the blurred image and multiplying by 80 (cv2.divide(original image, blurred image, scale=80).

(2) Contrast enhancement: Contrast-Limited Adaptive Histogram Equalization (CLAHE) was applied with a clip limit of 2 and a tile grid size of 10 × 10 to enhance image contrast.

(3) Binarization: A fixed threshold of 78 was applied (cv2.threshold(original image, 78, 255, cv2.THRESH_BINARY_INV)).

(4) Remove object: Connected-component analysis was used to remove small objects based on their size, defined by lower object size < 80 thresholds.

(5) Dilation: The binary image was dilated using a 4 × 4 kernel to reconstruct collapsed or discontinuous boundary line within each aggregate, thereby reinforcing individual contours.

(6) Erosion: A single erosion was applied using the same kernel to remove excessive peripheral regions introduced by dilation and prevent unintended merging between neighbouring aggregates.

(7) Dilation: A second dilation was subsequently performed using the same kernel to restore aggregate boundaries after erosion.

(8) Masking: The dilated image was masked with a 5,000 of radius to remove the well edges.

(9) Fill hole: Holes within aggregates were filled by replacing the value of 255 for the intensity value 0 of an object whose intensity value 0 is surrounded by 255.

(10) Remove frame overlap: Connected-component analysis was performed to remove any objects overlapping the image frame, based on image dimensions.

## Comparing apparent bulk density

Ten milligrams of microcarriers (3781; Corning), fibrillated chitosan nanofibers, fibrillated chitin nanofibers, and a chitosan/chitin nanofiber mixture (80:20) were each dispersed in 5 mL of water and transferred into a measuring cylinder. The suspensions were then allowed to settle under static conditions for 10 h. An image was acquired immediately after transfer (*t* = 0 h), and another image was acquired at *t* = 10 h.
